# Factors associated with platelet reactivity during dual antiplatelet therapy in patients with diabetes after acute coronary syndrome

**DOI:** 10.1038/s41598-020-59663-3

**Published:** 2020-02-21

**Authors:** Vacis Tatarunas, Nora Kupstyte-Kristapone, Vaidotas Zvikas, Valdas Jakstas, Remigijus Zaliunas, Vaiva Lesauskaite

**Affiliations:** 1 Institute of Cardiology of Lithuanian University of Health Sciences, Sukileliu 15, Kaunas, LT 50009 Lithuania; 2 Department of Cardiology of Lithuanian University of Health Sciences, Eiveniu 2, LT 50009 Kaunas, Lithuania; 3 Cardiovascular Center of Republican hospital of Siauliai, V. Kudirkos g. 99, 76231 Siauliai, LT Lithuania; 4 Institute of Pharmaceutical Technologies of Lithuanian University of Health Sciences, Sukileliu 13, Kaunas, LT 50009 Lithuania

**Keywords:** Predictive markers, Acute coronary syndromes, Diagnostic markers

## Abstract

Antiplatelet drugs are prescribed without considering the diabetic status of the patient. The objective of the current investigation was to determine the impact of clinical factors, CYP4F2 enzyme and 20-hydroxyeicosatetraenoic acid (20-HETE) concentrations on high on-treatment platelet reactivity in patients with diabetes treated with antiplatelet drugs following acute coronary syndromes. A total of 667 patients were included in the study. Dual antiplatelet drug loading dosages with aspirin (300 mg) and ticagrelor (180 mg) or clopidogrel (600 mg) were prescribed to all the studied patients. Testing of platelet aggregation was performed the day after loading antiplatelet drug dosages. Platelet aggregation test was done according to the classical Born method. Multivariate binary regression analysis demonstrated that insulin use and higher 20-HETE concentration increased the odds of high on-treatment platelet reactivity during the initiation of antiplatelet drug therapy (OR: 3.968, 95% CI: 1.478–10.656, *p* = 0.006 and OR: 1.139, 95% CI: 1.073–1.210, respectively, *p* < 0.001). Ticagrelor use decreased the odds of developing high on-treatment platelet reactivity (OR: 0.238, 95% CI: 0.097–0.585, *p* = 0.002). Data from this study revealed that high on-treatment platelet reactivity during dual antiplatelet therapy in patients with diabetes may depend on such factors as insulin prescription and 20-HETE concentration.

## Introduction

Diabetes may enhance the effect of risk factors involved in cardiovascular disease^1^. However, the treatment guidelines for acute coronary syndrome (ACS) recommend the same treatment strategy for both diabetic and non-diabetic patients^1,2,3^. Antiplatelet drugs are prescribed without considering the diabetic status of the patient^2^.

In the PLATelet inhibition and patient Outcomes (PLATO) study, the newer generation drug ticagrelor was demonstrated to be superior to clopidogrel in reducing ischaemic events in ACS patients, irrespective of whether they had diabetes^4^. More recent studies have reported that patients treated with clopidogrel had higher in-hospital mortality compared to those treated with prasugrel or ticagrelor^5^. Platelet aggregation studies also showed that patients with diabetes who use clopidogrel have higher platelet reactivity than patients without diabetes^6^. A critical meta-analysis done with 26 studies, which included 28.178 patients, recommended platelet reactivity testing in high risk patients (patients with diabetes, patients with multiple cardiovascular risk factors, and in patients with comorbidities)^7^.

Higher platelet reactivity in diabetic patients is due to increased platelet functions, including an increased response to stimulation by platelet aggregation agonists, adhesion to thrombogenic surfaces and platelet aggregation^8^. In general, oxidative stress and reduced antioxidant activity induced by hyperglycaemia significantly augment in diabetic patients, subsequently leading to platelet activation and hyperreactivity^9^. A recent study by Tunaru *et al*. showed that diabetes is also a disease of fatty acid metabolism^10^. Tunaru *et al*. showed that glucose induces the synthesis of 20-hydroxyeicosatetraenoic acid (20-HETE)^10^. 20-HETE is a CYP-derived eicosanoid, which is produced by CYP4A11 and CYP4F2 enzymes, usually found in the kidneys and the liver^11,12^. According to Tunaru *et al*., reduced 20-HETE formation leads to reduction of glucose-stimulated insulin secretion in pancreatic islets^10^.

Previous studies by our group showed that *CYP4F2* variants may significantly affect antiplatelet therapy^13–15^. However, we were unable to find a relationship of plasma CYP4F2 enzyme activity and 20-HETE concentrations on antiplatelet activity of drugs (in a relatively small sample of the patients, n = 146) during dual antiplatelet therapy (DAPT)^15^.

## Methods

The aim of the current investigation was to analyse the impact of clinical factors, CYP4F2 and 20-HETE concentrations on high on-treatment platelet reactivity in a sample of the patients representing a significant group of patients with diabetes treated with antiplatelet drugs ticagrelor or clopidogrel following acute coronary syndromes.

### Study population and inclusion criteria

**Figure 1:**
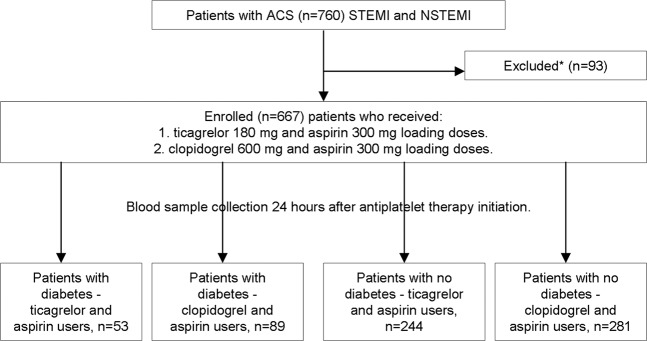
Study design. *Inclusion and exclusion criteria described in materials and methods section.

Clinical data and DNA samples of the patient population were collected during the SEN-09/2015 study^14^. All the patients were hospitalised for percutaneous coronary interventions (PCI) and stent implantation due to acute coronary syndromes (myocardial infarction or unstable angina) at the Department of Cardiology at the Lithuanian University of Health Sciences (LUHS) in Kaunas, Lithuania, from 2013 to 2017. Patient inclusion and exclusion criteria were described earlier^14^. Patients were divided into four groups according to their diabetic status (diabetic or non-diabetic) and the prescribed antiplatelet drug (Fig. 1): diabetic patients who were prescribed ticagrelor and aspirin, diabetic patients who were prescribed clopidogrel and aspirin, non-diabetic patients who were prescribed ticagrelor and aspirin, and non-diabetic patients who were prescribed clopidogrel and aspirin.

### Patient clinical data

A total of 667 patients were included in the study (Fig. 1). Dual antiplatelet drug loading dosages with aspirin (300 mg) and ticagrelor (180 mg) or clopidogrel (600 mg) were prescribed to patients before PCI, according to the European Society of Cardiology (ESC) guidelines^16^. Patients were prescribed either clopidogrel or ticagrelor irrespective of diabetes. Blood samples for the analysis were taken the next day, approximately 24 hours after loading of antiplatelet dosages.

All patients received standard treatment with statins, angiotensin-converting enzyme inhibitors (or angiotensin receptor I blockers) and β-adreno-blockers. Clinical characteristics of the patients were collected from the case histories.

### Platelet aggregation

Testing of platelet aggregation was performed as described above^15^, the next day after initiation of antiplatelet therapy. Classical Born method was used to perform platelet aggregation test. The intensity of light transmission in platelet rich and in platelet poor plasma was compared by using Chrono-Log 490–2D platelet aggregometer (Chrono-Log, Havertown, Pennsylvania, USA). Platelet aggregation was determined following induction with adenosine diphosphate (ADP; final concentration 5 μM; Chrono-Log, Havertown, Pennsylvania, USA), epinephrine (final concentration 10 μM; Chrono-Log, Havertown, Pennsylvania, USA) and arachidonic acid (ARA) (final concentration 0.5 mM; Chrono-Log, Havertown, Pennsylvania, USA).

### Detection of CYP4F2 and 20-HETE in blood plasma

Concentrations of the CYP4F2 enzyme and 20-HETE in blood plasma were measured as described above^15^. All plasma samples were tested in duplicate. The analysis of 20-HETE in blood plasma was performed at the Institute of Pharmaceutical technologies of the Lithuanian University of Health Sciences. Measurements of CYP4F2 enzyme were performed at the certified Laboratory of Molecular Cardiology. The microplate reader Stat-fax 4200 (Awareness Technology, Palm City, Florida, USA) with a primary wave-length of 450 nm was used. The intraassay precision of the CYP4F2 SEL399Hu kit is less than 10%, and the interassay precision is less than 12% as declared by the manufacturer of this kit. No significant cross-reactivity or interference between CYP4F2 and analogues was declared by the manufacturer. A group of healthy (n = 26) subjects (age range 29–90, median 54 years), consisting of 13 males and 13 females who did not have complains about their personal health, and were not taking antibiotics, antiplatelets and anticoagulants within the past 3 months, was also gathered in order to determine blood plasma CYP4F2 and 20-HETE concentration in healthy subjects.

### Statistical analysis

Platelet aggregation and concentrations of CYP4F2 and 20-HETE in blood plasma were evaluated using a nonparametric Kruskal–Wallis test. Fisher’s exact test and Pearson χ2 analysis were used for categorical variables. The relationship between the high on-treatment platelet reactivity after induction with ADP and patient clinical data was established using multivariate linear regression analysis. High on-treatment platelet reactivity was defined as platelet aggregation levels higher than 46% after induction with ADP^17^. All variables were chosen for the multivariable model by backward selection, with the final model containing only those with p < 0.05.

### Ethics approval and consent to participate

All the procedures used have been reviewed in compliance with the ethical standards of the Regional Bioethics Committee of Kaunas, Lithuania (the permission number is BE-2–42) and the World Medical Association Declaration of Helsinki on Ethical Principles for Medical Research Involving Human Subjects. Written informed consent was obtained from all the participants prior to inclusion in the study.

## Results

Among the patients, clopidogrel users were older as compared to ticagrelor users (range 39–89, median 68 years vs. range 29–90, median 64 years, *p* = 0.001). Patients with diabetes tended to receive clopidogrel more often than ticagrelor (24.1% vs 17.8%, *p* = 0.08). Also, patients with diabetes tended to be older than non-diabetic patients (range 37–86, median 69 years vs. range 29–90, median 66 years, *p* = 0.053). Female patients with diabetes received clopidogrel more often than non-diabetic female patients (Table 1). Female patients with diabetes that used clopidogrel were older than ticagrelor users (p = 0.043). Patients with diabetes had bigger body weight, body weight index and body waist circumference (Table 1). More non-diabetic patients with ST segment elevation myocardial infarction (STEMI) were prescribed ticagrelor. More patients with diabetes and recurrent myocardial infarction (reMI) were prescribed clopidogrel. Patients with diabetes were less frequently smokers (Table 1).

**Table 1:** Baseline patients’ sample description.

	Ticagrelor users			Clopidogrel users		
	Diabetes	No diabetes	*p*	Diabetes	No diabetes	*p*
	53 (100)	244 (100)		89 (100)	281 (100)	
Gender n (%)						
Males	42 (79.2)	179 (73.4)	0.487	54 (60.7)	202 (71.9)	0.049
Females	11 (20.8)	65 (26.6)		35 (39.3)	79 (28.1)	
Age in years median (min-max)	64 (38-82)	64 (29-90)	0.744	69 (40-86)	68 (39-89)	0.059
Males	63 (38-81)	62 (29-90)	0.167	66 (40-84)	63 (39-89)	0.26
Females	69 (43-82)	71 (43-88)	0.218	74 (60-86)	72 (56-88)	0.513
Patient weight in kg median (min-max)	95 (60-121)	84 (48-164)	<0.001	92 (47-158)	82 (45-132)	<0.001
Body weight index median (min-max)	31 (23-43)	28 (17-44)	<0.001	32 (16-50)	28 (17-55)	<0.001
Waist circumference median (min-max)	99 (81-130)	94 (63-122)	0.001	104 (68-137)	93 (69-128)	<0.001
ST-elevation MI n (%)						
STEMI	16 (30.2)	119 (48.8)	0.015	28 (31.5)	88 (31.3)	1
NSTEMI	37 (69.8)	125 (51.2)		61 (68.5)	193 (68.7)	
MI in anamnesis n (%)						
First	33 (62.3)	168 (68.9)	0.418	56 (62.9)	212 (75.4)	0.029
Recurrent	20 (37.7)	76 (31.1)		33 (37.1)	69 (24.6)	
Smoking n (%)						
Smokers	9 (17)	75 (30.1)	0.045	17 (19.1)	87 (31)	0.031
Non-smokers	44 (83)	169 (69.9)		72 (80.9)	194 (69)	
Concomitant drug users						
Diaprel n (%)	8 (15.1)	—	—	22 (24.7)	—	—
Glimepiride n (%)	3 (5.7)	—	—	2 (2.2)	—	—
Insulin n (%)	19 (35.8)	—	—	34 (38.2)	—	—
Metformin n (%)	33 (62.3)	—	—	60 (67.4)	—	—
ACE inhibitors n (%)	47 (88.7)	232 (95.1)	0.105	79 (88.8)	250 (88.9)	1
Angiotensin II						
receptor antagonists n (%)	5 (9.4)	11 (4.5)	0.175	12 (13.5)	28 (10)	0.335
Beta						
adrenoblockers n (%)	50 (94.3)	229 (93.9)	1	86 (96.6)	262 (93.2)	0.309
Statins n (%)	53 (100)	242 (99.2)	1	84 (94.4)	274 (97.5)	0.17
Spironolactone n (%)	12 (22.6)	60 (24.6)	0.86	23 (25.8)	62 (22.1)	0.472
Amiodarone n (%)	3 (5.7)	5 (2)	0.155	1 (1.1)	6 (2.1)	1
Ivabradin n (%)	7 (13.2)	24 (9.8)	0.46	20 (22.5)	42 (14.9)	0.105
Calcium Channel						
Blockers n (%)	8 (15.1)	11 (4.5)	0.009	11 (12.4)	21 (7.5)	0.192
Diuretics n (%)	20 (37.7)	50 (20.5)	0.012	41 (46.1)	71 (25.3)	<0.0001
Ranitidine n (%)	35 (66)	185 (75.8)	0.166	59 (66.3)	197 (70.1)	0.52
Proton pump inhibitors n (%)	7 (13.2)	18 (7.4)	0.175	2 (2.2)	5 (1.8)	0.675
Blood test parameters						
Platelet count (x10^9^/l)	210 (109-491)	228 (103-1000)	0.09	213 (94-422)	211 (101-796)	0.692
WBC (x10^9^/l)	9 (4-43)	9 (3-22)	0.788	9 (4-21)	8 (4-28)	0.206
C-reactive protein (mg/l)	9 (1-276)	4 (1-379)	0.115	5 (1-222)	4 (1-255)	0.064
Creatinine (µmol/l)	94 (53-262)	89 (53-303)	0.154	92 (47-478)	87 (48-220)	0.016
Hemoglobin (g/l)	132 (98-176)	136 (89-172)	0.437	137 (81-163)	139 (50-170)	0.143

WBC – white blood cells.

Calcium channel blockers were less frequently prescribed to ticagrelor users and non-diabetic patients. Patients with diabetes that used clopidogrel had higher creatinine levels and more frequently received diuretics.

Users of ticagrelor had higher platelet (range 103–1000, median 227 × 10^9^/l vs. range 94–796, median 211 × 10^9^/l, *p* = 0.001) and white blood cell (range 3–43, median 9 × 10^9^/l vs. range 4–28, median 8 × 10^9^/l, *p* = 0.012) counts when compared to clopidogrel users.

### Effect of antiplatelet activity in diabetic and non-diabetic patients

Platelet aggregation values with ADP were higher in clopidogrel users than in ticagrelor users (range 5–70, median 33%^Agr^ vs. range 5–77, median 20%^Agr^, *p* < 0.001). Higher platelet aggregation values were detected in diabetic patients who were prescribed clopidogrel compared to non-diabetic patients (*p* < 0.001), (Table 2). No such effect was found in ticagrelor users.

**Table 2:** Platelet aggregation levels, CYP4F2 and 20-HETE concentrations in diabetic and non-diabetic patients users of ticagrelor and clopidogrel.

	Ticagrelor users		*p*	Clopidogrel users		*p*
	Diabetes (n = 53)	No diabetes (n = 244)		Diabetes (n = 89)	No diabetes (n = 281)	
Platelet aggregation with ADP %^Agr^	21 (6–68)	20 (5–77)	0.186	39 (9–69)	31 (5–70)^*^	<0.001
Platelet aggregation with epinephrine %^Agr^	29 (6–74)	30 (4–94)	0.89	35 (10–92)	34 (6–89)	0.62
Platelet aggregation with ARA %^Agr^	13 (6–81)	13 (4–77)	0.484	14 (6–90)	16 (4–73)	0.593
	(n = 43)	(n = 197)		(n = 83)	(n = 233)	
CYP4F2 conc. ng/ml	14 (3–90)	12 (0–101)	0.096	6(0–88)	4 (0–132)	0.037
	n = 47	n = 208		n = 72	n = 226	
20-HETE ng/ml	8 (1–30)	6 (1–27)	0.006	6 (1–24)	6 (1–26)	0.983
	n = 33	n = 126		n = 48	n = 118	

### The impact of CYP4F2 and 20-HETE concentration in blood plasma on antiplatelet activity during initiation of dual antiplatelet therapy

Control sample with healthy subjects showed higher CYP4F2 concentrations in healthy subjects than in patients: range 6–81, median 15 ng/ml vs. range 0–132, median 9 ng/ml, *p* < 0.001, respectively. Levels of 20-HETE did not significantly differ in patients and in healthy subjects: range 1–30, median 6 ng/ml vs range 2–11, median 5 ng/ml, p = 0.133.

The CYP4F2 concentration was lower in clopidogrel users compared to ticagrelor users (range 0–132, median 5 ng/ml vs. range 0–101, median 12 ng/ml, *p* < 0.001). The CYP4F2 concentration (Table 2) was lower in non-diabetic patients that used clopidogrel compared to patients with diabetes (*p* = 0.037). Patients with diabetes, ticagrelor users tended to have higher CYP4F2 concentration than non-diabetic patients (Table 2).

Levels of 20-HETE were higher in patients with diabetes who were prescribed ticagrelor compared to non-diabetic patients (Table 2).

Patients with high on-treatment platelet reactivity had a lower CYP4F2 (range 0–122, median 6 ng/ml vs. range 0–132, median 9 ng/ml, *p* = 0.019), but a higher 20-HETE (range 1–28, median 10 ng/ml vs. range 1–30, median 6 ng/ml, *p* < 0.001) concentration than patients with a good response to antiplatelet therapy.

### Binary logistic regression model

Multivariate binary regression analysis (Table 3) demonstrated that insulin use and a higher 20-HETE concentration all increased the odds of high on-treatment platelet reactivity during the initiation of antiplatelet therapy. Ticagrelor use decreased the risk of developing high on-treatment platelet reactivity (Table 3).

**Table 3:** Multivariate binary regression analysis of the association between clinical factors and high on-treatment platelet reactivity.

Variable	Odds ratio	95% CI	*p*
Insulin use	3.968	1.478–10.656	0.006
Ticagrelor use	0.238	0.097–0.585	0.002
20-HETE (ng/ml)	1.139	1.073–1.210	<0.001

## Discussion

The results of this study demonstrated that CYP4F2 and 20-HETE may have a significant role in patients with diabetes and in users of dual antiplatelet therapy. For the first time, the results revealed that patients with high on-treatment platelet reactivity had a higher 20-HETE but a lower CYP4F2 concentration in blood plasma. Patients users of clopidogrel had a lower CYP4F2 concentration than ticagrelor users. Healthy subjects had a higher CYP4F2 concentration than our studied patients. Also, patients with diabetes, users of ticagrelor, had a higher 20-HETE concentration than non-diabetic patients.

Despite research data showing decreased activity of clopidogrel in diabetics, there are currently no guidelines which recommend different strategies for patients with diabetes^2,3^. Patients in the present study who were prescribed ticagrelor were younger and more frequently had STEMI. Patients with recurrent MI (in anamnesis) and diabetes were more frequently prescribed clopidogrel. Patients with diabetes tended to be older than non-diabetic patients. In addition, female patients with diabetes were less frequently prescribed ticagrelor than non-diabetic females. The rate of clopidogrel prescription to diabetic patients observed in our study confirms the observations of other researchers, who emphasise that factors such as diabetes have no decisive significance in clinical practice when choosing a more potent antiplatelet drug than clopidogrel^1,18,19^. Diabetes may, however, aggravate other risk factors involved in cardiovascular disease, especially in women^1^.

It is noteworthy that our presented patients users of ticagrelor had elevated platelet and white blood cells counts. Patients with diabetes users of clopidogrel had higher creatinine level and more frequently received diuretics. Low lymphocyte count is a poorer prognostic marker during STEMI, as it correlates with inflammatory process during acute myocardial infarction. Low lymphocyte count during first 96 hours of a STEMI might predict recurrent myocardial infarction^20^. Studies also showed that low glomerular filtration rate might be associated with worse outcomes in ACS STEMI and non-ST-segment elevation myocardial infarction (NSTEMI) patients^21^.

The PLATO study, conducted about a decade ago, showed that ticagrelor is more effective for reducing ischaemic events in diabetic patients with ACS when compared to clopidogrel^4^. PLATO study results also showed, that diabetes has no effect on treatment outcomes during ticagrelor therapy^4^. However, Nardin *et al*., in a small patient sample (n = 224), recently showed that diabetic patients who used ticagrelor had a higher rate of platelet reactivity than non-diabetic patients^22^. Our results are in line with those of the PLATO study. Diabetes had no effect on antiplatelet treatment with ticagrelor, but significantly reduced the antiplatelet effect of clopidogrel. Ticagrelor is a newer generation, faster onset antiplatelet drug than clopidogrel. It acts as a reversible non-competitive P2Y12 receptor inhibitor, and binds distinct from the ADP binding site in the P2Y12 receptor. Clopidogrel, on the contrary, binds directly at the binding site of ADP and is its competitive inhibitor. Ticagrelor does not require metabolic activation^23,24^. By contrast, clopidogrel requires metabolic activation, and its therapeutic effect is highly dependent on hepatic cytochrome P450 enzyme activity (mainly CYP2C19). Variants of *CYP2C19* have a critical effect during clopidogrel therapy^25,26^. The therapeutic effect of clopidogrel is also influenced by other factors, which do not usually affect treatment with ticagrelor^14^. Ticagrelor has a pleiotropic activity. It inhibits the reuptake of adenosine by red blood cells, thereby elevating blood plasma adenosine concentrations^27^. Elevated adenosine concentrations might reduce platelet aggregation by acting on low-affinity platelet A_2A_ receptors^27^. In addition, adenosine dilates coronary arterioles in humans^28^.

In our earlier investigations, we found that *CYP4F2* gene variants may significantly affect antiplatelet therapy during clopidogrel or ticagrelor treatment after ACS^13,14^. In the current study, we performed a more profound analysis in a larger sample of patients. Data from the current investigation show higher CYP4F2 concentration in blood plasma of healthy subjects than in patients. Also, similar CYP4F2 concentrations are found in ticagrelor users but are lower in blood plasma of clopidogrel users. Most of cytochromes P450 are suppressed during inflammation by proinflammatory cytokines, eicosanoids and histamine^29,30^. It is noteworthy that CYP4F2 is usually induced during inflammation^31,32^, as it performs ω-hydrolysis of leukotriene B4 or arachidonic acid^33^, and it plays a critical role in resolution of inflammation^31,32^. Patients with diabetes users of clopidogrel have a higher CYP4F2 concentration than non-diabetic patients. Also, patients with diabetes users of ticagrelor tended to have a higher concentration of CYP4F2 in blood plasma than non-diabetics. Higher concentration of CYP4F2 in patients with diabetes might be associated with higher extent of inflammatory processes as compared to the patients without diabetes. Higher CYP4F2 concentration in plasma of ticagrelor users might also be determined by pleiotropic effects of ticagrelor^27^. Ticagrelor may participate in resolution of inflammation in patients with ACS. However, the first limitation of this study is that we did not evaluate the activity of CYP4F2, as we only detected the concentration of this enzyme. Another limitation is that we did not determine the origin of this enzyme. CYP4F2 is an enzyme usually found in endoplasmic reticulum. However, under stress conditions, it may be secreted with extracellular vesicles into the blood stream^34^.

20-HETE is a metabolite of arachidonic acid, which is formed by hepatic cytochrome P450 ω-hydroxylases. The two major enzymes that produce 20-HETE in humans are CYP4F2 and CYP4A11^35^. In general, the CYP4F2 enzyme is usually expressed in the kidney and liver. CYP4A11 is found in the S2 and S3 segments of the proximal renal tubules^11,12^. Our results showed that concentration of 20-HETE was higher in patients with diabetes receiving ticagrelor as compared to non-diabetic patients. The most interesting is that our presented patients with high on-treatment platelet reactivity had elevated 20-HETE, but a lower CYP4F2 concentration. CYP4F2 may represent the resolution of inflammation in the body (as it was described already), on the contrary, blood plasma 20-HETE may reflect dysregulation of inflammatory or metabolic processes. According to *Li et al*.^36^, 20-HETE plays a key role in insulin resistance, endothelial dysfunction, cardiovascular disease and diabetes pathogenesis. 20-HETE inhibit insulin-stimulated nitric oxide synthase (NOS) function and the production of nitric oxide (NO) in human umbilical vein endothelial cells (HUVECs). It is also associated with altered tyrosine phosphorylation of insulin receptor substrate-1 (IRS-1) and subsequent activation of PI3-kinase (PI3K), resulting in defective activation of Akt/eNOS signalling pathway^36^. Recently, Tunaru *et al*., showed that 20-HETE is an endogenous regulator of insulin secretion in pancreatic β-cells via a direct effect on Free fatty acid receptor 1 (FFAR1) receptors^10^. Inhibition of 20-HETE production or FFAR1 receptor blockage was associated with a reduction in insulin secretion, and 20-HETE, in turn, was shown to be produced as a response to high glucose levels^10^.

We performed a multivariate binary regression analysis to identify factors that significantly affect the risk of high on-treatment platelet reactivity during dual antiplatelet therapy. According to Bonello *et al*., high on-treatment platelet reactivity in patients treated with antiplatelets after PCI, was defined as higher than 46% of maximal 5-μmol/l ADP-induced aggregation^17^. Our results showed that insulin use and a higher 20-HETE concentration were all found to increase the risk of high on-treatment platelet reactivity. In contrast, ticagrelor use reduced the odds for high on-treatment platelet reactivity. In our patient sample, insulin was prescribed only to diabetic patients to control blood glucose levels. In clinical practice, insulin is prescribed to patients with type 1 diabetes, and also to patients with type 2 diabetes who are resistant to the action of insulin^37^. Diabetic patients usually have higher platelet reactivity, elevated oxidative stress and higher circulating free fatty acids (FFA), associated with chronic inflammation and a prothrombotic state^8,9,38–40^. Because of this, it is possible that insulin injection may have an effect on 20-HETE release.

Data from this study revealed that the efficiency of antiplatelet therapy may depend on such factors as insulin prescription and 20-HETE concentration. This study has some limitations. Firstly, the clinical factors may not be sufficiently reflected in our study. Blood glucose, glycated haemoglobin, and blood plasma lipoprotein levels were not analysed, and we did not evaluate the impact of *CYP2C19* and *CYP4F2* variants on the antiplatelet effect of drugs. Blood glucose levels, except patients with diabetes, are not routinely measured in our hospital. Patients with acute coronary syndromes usually have lower total cholesterol and high-density lipoprotein cholesterol levels. Low-density lipoprotein cholesterol also decreases during MI^41^. A significant portion of our described patients previously received lipid-lowering drugs. Thus, due to several factors, lipid profile might not be presented correctly in our studied patients.

## Conclusions

Our results demonstrated that insulin use and 20-HETE level may determine high on-treatment platelet reactivity in patients receiving DAPT after ACS. The results of this study also showed that diabetes had no effect on antiplatelet treatment with ticagrelor, but it significantly reduced the antiplatelet activity of clopidogrel. Specific attention must be given to managing women with diabetes.

## Data Availability

The datasets and resources generated during and/or analyzed during the current study are available from the corresponding author upon reasonable request.
